# Patients’ Perspective on Participation in Care With or Without the Support of a Smartphone App During Radiotherapy for Prostate Cancer: Qualitative Study

**DOI:** 10.2196/mhealth.6829

**Published:** 2017-07-26

**Authors:** Maria Hälleberg Nyman, Catharina Frank, Ann Langius-Eklöf, Karin Blomberg, Kay Sundberg, Yvonne Wengström

**Affiliations:** ^1^ School of Health Sciences Örebro University Örebro Sweden; ^2^ Department of Neurobiology, Care Sciences and Society Karolinska Institutet Stockholm Sweden

**Keywords:** patient participation, prostate cancer, radiotherapy, smartphone

## Abstract

**Background:**

Patients with prostate cancer are often cared for as outpatients during radiotherapy, which can be an aggravating circumstance for patient participation. There is a need to evaluate whether an interactive smartphone app could enable participation in care, specifically during treatment for prostate cancer. The interactive app (Interaktor) used in this study is developed in codesign with patients and health care professionals; it includes daily reports of symptoms, a risk assessment model, evidence-based self-care advice, along with the provision of immediate access to clinicians.

**Objective:**

The aim of this study was to explore how patients with prostate cancer perceived their participation with or without the support of the smartphone app during radiotherapy.

**Methods:**

A total of 28 prostate cancer patients receiving adjuvant radiotherapy were interviewed about their perceived participation in their own care. All the patients interviewed in this study participated in an intervention study where the control group received standard care that comprised having access to a contact nurse to turn to with any concerns during their treatment. In addition to standard care, the patients in the intervention group received the app downloaded in a smartphone. The patients’ age ranged between 57 and 77 years; 17 patients used the smartphone app. The interviews were analyzed with directed qualitative content analysis.

**Results:**

The four dimensions of patient participation, which include mutual participation, fight for participation, requirement for participation, and participation in getting basic needs satisfied, were confirmed as valid perspectives in the interviews with the patients with prostate cancer, irrespective of whether they used the smartphone app. However, the patients who had used the smartphone app described it as a facilitating factor, especially for mutual participation.

**Conclusions:**

Using innovative ways to communicate with patients, such as an interactive app for symptom management with contact with health care in real time, can successfully help achieve increased patient participation in care.

## Introduction

Advancements in the area of mobile smart devices (phones and tablets) have dramatically influenced the role of technology in health care [1,2]. There is now a range of various mobile apps available that differ in many respects, including their level of interactivity, evidence-based content, and role in the health care process [1,2]. The future challenge is to improve remote monitoring and to embed the technology in the human-executed processes [1]. Many interactive apps focus on self-management activities carried out by patients during the cancer care treatment period, but only a few address supportive care for cancer after the treatment is completed [1,3].

It is emphasized that care and support for patients affected by cancer should focus on recovery, personalized care-planning, support for self-care, early recognition of signs and symptoms for further disease, and routine use of patient-reported outcome measures (PROM) [4]. A patient-reported outcome is defined as “any report of the status of a patient’s health condition that comes directly from the patient, without interpretation of the patient’s response by a clinician or anyone else” [5,6]. In a review, studies indicate that routine clinical use of PROM may improve early identification and recognition of symptoms, as well as the communication between patients and health care staff [7]. The incorporation of PROM into clinical practice may support patients in becoming active in self-care and may enhance early identification of appropriate interventions [7-9].

The concept of self-care is described as a central part of patient participation [10], and over the past 10 years, there has been a shift in health care delivery with a general move toward supporting patients to engage in all forms of self-care. Furthermore, patients are expected to take increased responsibility for and participate in their own care [11], although different patients want different levels of participation [12]. Most patients express that they want to participate in their care process, but around one-third want to stay passive [12]. However, most patients do not achieve their desired role [12].

For men diagnosed with prostate cancer, patient participation has been explored in situations related to the choice of medical treatment [13-15], and the results show that most men prefer active involvement in their prostate cancer treatment decisions. Studies about self-care during treatment are scarce, but patients perceive that waiting for health care staff to make contact and being given incomplete information about symptoms and self-care is distressing [16]. During treatment, patients affected by prostate cancer are often cared for as outpatients, which places further demands on both the patients, by expecting them to be experts on their own health, and on the health care staff, in terms of providing a suitable context for the planning, provision, and assessment of individualized care [17].

Therefore, in collaboration with Health Navigator, a company that specializes in new innovative care solutions, we developed an interactive app (Interaktor) for use in smartphones or tablets for the reporting and managing of symptoms during radiotherapy for patients with prostate cancer [16,18]. The app includes PROM in that the symptom assessment is completed by the patient with immediate transmission of the results to a designated health care professional and using a risk assessment model based on symptom occurrence and frequency, the app sends alerts by text messages (short message service) if any symptom assessments are of concern. Furthermore, the app offers access to evidence-based self-care advice related to the reported symptoms, links to relevant websites for more information, and provides access to the symptom history presented in graphs over time as well as an open comment section. The content was developed in a process of codesign with patients and staff and with support from the literature [16], and the app has been found to be feasible and useful [18]. Previous research has shown that to achieve high uptake and interactivity with technology, it is important to involve patients in the development process to ensure that the content is relevant and usable to them [19,20]. In addition to receiving standard care, the patients submit daily reports throughout the treatment period and over the following 3 weeks [16,18]. When testing interventions that include mobile technologies, it is important to evaluate their use from the patient’s perspective [21,22]. In our research program, we hypothesize that the use of mobile technology may contribute to early detection of symptoms and side effects within cancer care, thereby aiding prompt management and increasing patients’ perceptions of participatory care. Therefore, the aim of this study was to explore how patients affected by prostate cancer perceive patient participation during radiotherapy treatment with or without the support of the mobile app (Interaktor).

## Methods

### Design

This study is a part of an experimental study conducted at two university hospitals, which included patients who had been diagnosed with prostate cancer. Ethical approval was obtained from the Regional Ethical Review Board of Uppsala, Sweden (reference number 2011/256). The intervention group that used the app, Interaktor, during radiotherapy treatment, was compared with a historical control group with data collected in the immediate period before the intervention implementation [23]. The patients who used the app during radiotherapy reported less symptom burden than those who did not use the app [23]. A descriptive qualitative design with a directed approach [24] was chosen to increase the understanding of the patients’ perceptions of participation in care and whether their perception was related to using the app or not. The applied methodological theoretical foundation included an inductive approach for data collection and a deductive approach for analyzing the interviews; the theoretical underpinnings of the qualitative descriptive research design were drawn from the general tenets of naturalistic inquiry [25].

### Participants

The participants were patients diagnosed with prostate cancer receiving adjuvant radiotherapy (external and internal radiation) for 8 to 11 weeks at two university hospitals (one rural and one urban) in Sweden. A purposive sampling strategy from both groups was adopted by using a sampling frame [26] to capture a range of patient characteristics, including their age, area of residence, and whether they had used the smartphone app, Interaktor, during the treatment period. Thirty-two patients were asked to partake in the interview study. Altogether, 28 patients agreed to participate, of which 17 patients used the smartphone app. Their age ranged between 57 and 77 years; 13 patients were living in rural/suburban areas and 15 in urban areas. Table 1 shows an overview of the sociodemographic and clinical characteristics of the participants in the study.

**Table 1:** Sociodemographic and clinical characteristics for patients with prostate cancer (N=28) included in the smartphone app and standard care groups.

Variable		Smartphone app group (n=17)	Standard care group (n=11)
**Age, in years**			
	Mean (SD)	70 (4.0)	70 (5.4)
	Median (range)	71 (63-76)	71 (57-76)
**Living situation, n (%)**			
	Married/living with partner	12 (70)	9 (82)
	Living alone	3 (18)	2 (18)
	Other	2 (12)	0
**Area of living, n (%)**			
	Rural/Suburban	9 (53)	4 (36)
	Urban	8 (47)	7 (64)
**Educational level, n (%)**			
	Junior compulsory	2 (12)	6 (55)
	Senior high school	6 (35)	2 (18)
	Postgraduate/University	8 (47)	2 (18)
	Missing	1	1
**Occupation, n (%)**			
	Working	2 (12)	1 (9)
	Retired	12 (72)	10 (91)
	Other	2 (12)	0
	Missing	1	0
**Clinical T stage, n (%)**			
	1	4 (24)	3 (27)
	2	7 (41)	6 (55)
	3	3 (18)	2 (18)
	Missing	3	0
**Gleason, n (%)**			
	6	1 (6)	2 (18)
	7	6 (35)	5 (46)
	8	4 (24)	3 (27)
	9	6 (35)	1 (9)
**Type of radiotherapy treatment, n (%)**			
	External beam radiotherapy (EBRT)	6 (35)	2 (18)
	Brachytherapy combined with EBRT	11 (65)	9 (82)
**Additional treatment, n (%)**			
	Adjuvant hormonal therapy^a^	12 (71)	7 (64)

^a^All patients received radiotherapy, and a majority received hormonal therapy in addition to radiotherapy.

### Study Procedure

Standard care during radiotherapy comprises regular contact with therapy staff and access to a contact nurse regarding any treatment-related concerns. No regular medical support or standard procedures are included in the care during the treatment period. The patients in the intervention group received standard care and were provided with a smartphone with the app, Interaktor, installed and instructed to answer the symptom assessment (frequency and distress of 14 symptoms) daily, during office hours on weekdays during the radiotherapy period, and 3 weeks after. The patients were given thorough instructions by the researchers initially on how to use the smartphone app (assessment, connection to self-care advice (n=12), and graphs). In addition, they were given a written checklist, including a phone number for technical support. The patients were given an individual log-in and personal identification number code to get access to the app. They were also informed that in case of an alert, a study-specific nurse would call them during office hours and that acute problems occurring at other time points had to be handled according to the standard procedure of the oncological clinic. The patient’s self-report was directly sent via the secure server accessible from a Web interface for the study-specific nurses at the hospitals. The patients in the control groups received standard care only.

### Data Collection

To gain an understanding of the patients’ perceptions of their participation in care during their treatment, open-ended interviews [27] were conducted approximately 5 weeks after completion of radiotherapy (ie, 2 weeks after their final report was made in the app for the patients who had used the app). Researchers with previous experience of conducting patient interviews carried out the interviews. The same question was posed to all participants: *“Can you tell me about the time when you went for your treatments—how did you perceive your participation in the care during the treatment period?”* They were encouraged to speak as freely as possible, and if the word “participation” was difficult for them to understand, it was explained using synonyms such as “involvement” and “partaking.” Follow-up prompts such as *“Please tell me more about that”* or *“Can you give an example?”* were included in the interview when needed.

The interviews were all audio-recorded with the participants’ permission; they lasted between 30 and 60 min, and according to patients’ preferences, were held either at their homes, in the hospital, or in a private room at the university.

### Data Analysis

The analyses were guided by the principles proposed by Hsieh and Shannon [24] and assumed a qualitative directed approach. An analyzing scheme based on the dimensions of patient participation from the patients’ and health care providers’ perspectives, developed for use in qualitative studies by Frank et al [28,29], was chosen. The four dimensions employed were: *Mutual participation*—which describes when patients have requirements, for example, time and respect, and when the patient encounters health care staff in a mutually shared dialogue; *Fight for participation*—which represents the patients’ own struggle for participation; *Requirement for participation*—which includes the necessary elements for gaining mutual participation, for example, time and information; and *Participation in getting basic needs satisfied*—which includes participation in terms of getting basic needs such as nutrition, and pain and worry, satisfied without requests from the patient [30,31].

The interviews were first transcribed verbatim, and all the transcripts were read repeatedly to obtain a sense of the data as a whole. Next, the interviews with the patients in the intervention group and the interviews with the patients in the control group were analyzed separately from the authors in pairs of 2 to manage the extensive dataset and to increase trustworthiness. The authors individually divided the text into meaning units in agreement with the study’s purpose. The meaning units were then discussed between all of the authors, condensed, and coded carefully, while keeping the essence of the statements made by the patients. In the next step, the codes were sorted into groups based on the analyzing scheme outlined by Frank et al [30,31], but allowances were also made for the inclusion of emergent dimensions that might reflect patient participation. All of the authors critically reviewed each step in the analytical process to achieve trustworthiness. Selected quotations are presented to illustrate the findings. Microsoft Word was used as a tool to organize data throughout the entire analysis process.

## Results

The descriptions of participation among the patients are presented on the basis of the four dimensions of patient participation, with no additional dimension of patient participation having emerged in the analysis of the interviews.

### Mutual Participation

In general, in both groups *Mutual participation* was described when both parties—patients and health care staff—were actively involved in a dialogue in some way. During an encounter, it was important that both parties listened and asked questions. When the patients actively contacted health care providers, the health care staff were described as having met the patients’ needs.

*Mutual participation* was more prominent in the group of patients who had the smartphone app. They perceived participation when they reported symptoms in the app and when they received a response from the nurse if the symptom report generated an alert and had a dialogue about how to resolve the problem. The patients knew that there was a nurse present to receive their reports, and they did not have to search for the right health care staff to pose their questions to:

In those cases, the app is a point of contact. I know that there is someone who gets a notice on their screen that shows “he has a problem right now,” and they get in touch. It’s really good.

Patient with smartphone app

Some of the patients using the smartphone app described how they also appreciated having the opportunity to send a personalized response via the app, and thereby communicate with the nurse and obtain a response:

I reported that I felt feverish and dizzy one day and then someone called me up. I talked to the nurse and she confirmed that it wasn’t anything serious.

Patient with smartphone app

The smartphone app was described as a security line and as a link to someone who was caring for you and being in control of the situation:

The smartphone application feels very secure for me and if you have a problem, then you can indicate that and a nurse will call you...so it’s like having health care staff in your house.

Patient with smartphone app

### Fight for Participation

In the category, *Fight for participation*, the patients’ descriptions were similar in both groups. Patients described that they sometimes had to fight to get an answer regarding their concerns at the clinic. Some patients even described that they had to fight to get the care they found themselves in need of.

Patients described adopting different strategies for participating in their own care process. One strategy was to make phone calls to various health care units involved in the care process. Another strategy was to pursue one specific health care staff member using repeated attempts at participation. One patient described how he tried to get in contact with his contact nurse by calling her on the phone on repeated occasions. During the process of radiotherapy, patients received outpatient treatment, and they frequently had questions about new symptoms, medications, and a need for someone to talk to. Some patients experienced feeling frustrated that they had unanswered questions, and they did not know where to get answers. They expressed that the health care staff in the radiation department were only able to answer questions relating to the radiotherapy, but not other questions regarding their care and illness:

...yes, the health care staff who provide the radiotherapy aren’t able to respond to any questions. They can’t. If I ask them about my urinary problems, they tell me I have to go to the inpatient clinic. They don’t have the knowledge, they’re just doing their own thing.

Patient with smartphone app

Some patients without the smartphone app searched on the Internet to obtain more information to get answers to their questions:

...in the beginning I was on the Web and looked around trying to read a little here and there, but there is a huge amount to read, I just read a little bit from a few of all the web sites.

Patient without smartphone app

Another strategy they adopted was when patients perceived that relatives could provide some support in their struggle to become involved. If patients themselves were unable to hear and understand the information they received from the health care staff, relatives provided support in listening to conversations between the health care staff and the patient to gain information that may be relevant to their situation:

Luckily I had my wife with me, who provided another pair of ears to listen with, to pick up what I didn’t hear or understand, and we helped each other to summarize. But it shouldn’t be like that.

Patient without smartphone app

### Requirement for Participation

*Requirement for participation* was generally described in both groups in terms of receiving clear information in advance, both verbally and written. Patients perceived staff as being pleasant and having professional competence, especially in relation to continuity of health care. When the health care staff took the initiative to establish contact, the patients felt welcomed and respected.

The participants described how the health care staff had clearly described the radiotherapy routine so that the patients would know what would happen next. The patients perceived that they were involved in the process of radiotherapy by being given information in different forms. However, patients perceived that the health care staff set the conditions for when and how the participation would take place and in what form. The basis of contact was focused on the implementation of the aspects in the care process rather than on the patient as a person. The opportunity to have influence on when the radiotherapy appointment should take place was also expressed as a requirement for participation.

Patients using the smartphone app described the app as a device that enhanced their perceived participation. They expressed that the content of self-care advice and the weblinks in the app promoted their participation in their care:

Yes, that was really good [self-care advice]. In some circumstances you felt, “Should it really be like this?” There was information there, so that was good. I’ve used it and looked at it.

Patient with smartphone app

### Participation in Getting Basic Needs Satisfied

The patients described experiencing *Participation in getting basic needs satisfied* in similar ways in both groups. Patients gave examples of getting help as being given prescriptions for antibiotics or analgesia and also in being prescribed care and treatment for complications related to radiotherapy:

Well, I told the doctor that I found it difficult to pee. Then he prescribed a pill. And now am I taking it every night, and well, it is better now.

Patient without smartphone app

However, for the patients who had used the smartphone app, sometimes a medication prescription had been communicated by reporting symptoms in the app. On the following day, when they attended the clinic for their radiotherapy session, a prescription had already been prepared.

Basic needs were satisfied when the staff offered meals and helped to arrange transportation. Long-distance patients also received help with sorting out their accommodation at the patient hotel when they needed it.

Patients, who had received brachytherapy in addition to radiotherapy were cared for as inpatients during brachytherapy, described having severe urinary problems during that period, and how they spontaneously, without asking, received help with urinary catheterization or medication for urgency incontinence:

During night-time the urine flows into the bag and they’re supposed to continuously check that the bag doesn’t get full; they almost come tiptoeing on the floor with flashlights so as not to disturb the patients that are sleeping—very touching.

Patient with smartphone app

## Discussion

### Principal Findings

The findings indicate that the support of a smartphone app could enhance patients’ experiences of being in close and continuous contact with health care services throughout the treatment process for prostate cancer. The patients who used the smartphone app, Interaktor, more commonly described experiences of participation as being mutual than those not using the app. These patients also described that they felt active, took their own initiatives, and had the opportunity to express their problems and concerns. Overall, patients in both groups described that their requirements for participation were met when they encountered health care staff that met them on equal terms in a pleasant and professional manner with high levels of competence. The patients with the smartphone app, Interaktor, experienced this also when reporting symptoms in the app and getting a call back from the nurse. This kind of passive receiving of care has also been described as participation in that the patients accept and accordingly resign themselves to receiving care without taking up the possibility to engage in active participation [32]. Participants in our study explicitly stated that they wished to participate on an individual basis. They also expressed that the health care staff in the radiotherapy departments responsible for the radiation treatment only responded to concerns in that specific area of care, and patients lacked a stable contact to help them navigate through the illness trajectory.

It is evident that patients cannot be treated as a homogenous group; they have different needs and wishes for participation [33,34]. Mutual participation could be developed within different forms of communication; for example, face-to-face, using a traditional telephone, or using the smartphone app for reporting and managing symptoms. If patient participation is to occur in the health care setting, there is a crucial need for establishing a relationship [10] built on the patient’s perspective [28,35]. Patients who used the smartphone app, Interaktor, experienced a personal relationship with the nurse on the receiving end, despite having only brief contact. It is evident that patient participation does not require extended conversations as has also been previously described [36]; the patients who had the smartphone app also felt safe knowing that there was someone who would immediately respond to their needs.

It is important to highlight that factors such as age, gender, race/ethnicity, education, level of income, marital status, employment status, socioeconomic status, type and stage of cancer, and the patient’s health status may influence role preferences in participation [34,37-39]. Other factors that inhibit participation include lack of time, poor interaction between different parties, lack of staff resources and high staff turnover [40]. However, it is not possible to predict which patients will prefer passive, active, or collaborative roles in participation in their care [13,41]. In care where patients perceive high participation in their own care, it has been shown that there is a higher quality of care, fewer mistakes, and a more positive image of the health care organization [42]. If the aim is to truly achieve patient participation, a more holistic and individualized approach is necessary for this to occur in the health care setting [43]. Moreover, applying patient participation in care prevents health care staff from imposing care that patients otherwise may not want [44]. Our app, Interaktor, seems to facilitate participatory care by engaging patients to report symptoms daily during treatment, regularly view their symptoms in graphs, and read self-care advice, and at the same time, by making patients feel secure that a nurse calls if a symptom report is alarming. Thus, the use of mobile health (mHealth) facilitates new ways to communicate with patients and may in the long run have an impact on how health care as a whole is organized [45].

### Limitations

A potential limitation of this study is the participants’ understanding of the concept of patient participation. When performing the interviews, this aspect was taken into consideration; however, some of the participants had difficulties expressing the ways in which they had participated or not in their care, apart from the decision of whether to be treated with surgery or radiation therapy. Furthermore, using a predetermined analysis scheme may have influenced the interpretation of the data. The four dimensions of patient participation employed were originally developed in an emergency context, and there was a potential risk that the dimensions would not be applicable to patients undergoing treatment for prostate cancer. However, we found that the wordings for these dimensions were not expressed within a specific context and found the framework to be suitable for the patient group in this study. Also, the suitability of this framework is supported by studies performed in other contexts relating to patient participation that show similar results [35,36,40,46].

Another potential limitation is that the distribution of the educational level and Gleason scores were not the same among the interviewed patients in the smartphone app group and the standard care group. These factors were not taken under consideration in advance, and the patients were selected to achieve a variation in age, area of residence, and whether they had used the smartphone app, Interaktor, during the treatment period, which we assumed could have an impact on the patients’ experience of participation during their radiotherapy treatment period. Earlier research has shown that lower education levels is associated with lower health literacy [47,48], which is an important parameter to consider in further development of mHealth.

### Conclusions

Innovative ways of communicating with prostate cancer patients in purposeful, short interactions, including the provision of supportive care by giving advice using smartphone apps, can shape patients’ perceived participation in their care.

## Abbreviations

EBRT: external beam radiotherapy
mHealth: mobile health
PROM: patient-reported outcome measures
